# Reasons for not reaching or using web-based self-management applications, and the use and evaluation of Oncokompas among cancer survivors, in the context of a randomised controlled trial

**DOI:** 10.1016/j.invent.2021.100429

**Published:** 2021-07-15

**Authors:** A. van der Hout, C.F. van Uden-Kraan, K. Holtmaat, F. Jansen, B.I. Lissenberg-Witte, G.A.P. Nieuwenhuijzen, J.A. Hardillo, R.J. Baatenburg de Jong, N.L. Tiren-Verbeet, D.W. Sommeijer, K. de Heer, C.G. Schaar, R.J.E. Sedee, K. Bosscha, M.W.M. van den Brekel, J.F. Petersen, M. Westerman, J. Honings, R.P. Takes, I. Houtenbos, W.T. van den Broek, R. de Bree, P. Jansen, S.E.J. Eerenstein, C.R. Leemans, J.M. Zijlstra, P. Cuijpers, L.V. van de Poll-Franse, I.M. Verdonck-de Leeuw

**Affiliations:** a Department of Clinical, Neuro- and Developmental Psychology, Amsterdam Public Health Research Institute, Vrije Universiteit Amsterdam, the Netherlands; b Cancer Center Amsterdam (CCA), Amsterdam UMC, Amsterdam, the Netherlands; c Amsterdam UMC, Vrije Universiteit Amsterdam, Department of Otolaryngology – Head and Neck Surgery, Amsterdam, the Netherlands; d Amsterdam UMC, Vrije Universiteit Amsterdam, Department of Epidemiology and Data Science, Amsterdam, the Netherlands; e Department of Surgery, Catharina Hospital, Eindhoven, the Netherlands; f Department of Otolaryngology and Head and Neck Surgery, Erasmus MC Cancer Centre, Erasmus Medical Center, Rotterdam, the Netherlands; g Department of Hematology, Erasmus Medical Center, Rotterdam, the Netherlands; h Department of Internal Medicine, Flevoziekenhuis, Almere, the Netherlands; i Amsterdam UMC, University of Amsterdam, Department of Medical Oncology, Amsterdam, the Netherlands; j Amsterdam UMC, University of Amsterdam, Department of Hematology, Amsterdam, the Netherlands; k Department of Internal Medicine, Gelre ziekenhuis, Apeldoorn, the Netherlands; l Department of Otolaryngology, Head and Neck Surgery, Haaglanden MC, The Hague, the Netherlands; m Department of Surgery, Jeroen Bosch Ziekenhuis, Den Bosch, the Netherlands; n Department of Head and Neck Surgery, Netherlands Cancer Institute, Amsterdam, the Netherlands; o Department of Hematology, Northwest Clinics, Alkmaar, the Netherlands; p Department of Otorhinolaryngology – Head and Neck Surgery, Radboud University Medical Center, Nijmegen, the Netherlands; q Department of Hematology, Spaarne Gasthuis, Hoofddorp, the Netherlands; r Department of Surgery, St. Anna Hospital, Geldrop, the Netherlands; s Department of Head and Neck Surgical Oncology, Utrecht University Medical Center, Utrecht, the Netherlands; t Department of Surgery, Elisabeth-TweeSteden Hospital, Tilburg, the Netherlands; u Amsterdam UMC, Vrije Universiteit Amsterdam, Department of Hematology, Amsterdam, the Netherlands; v Department of Research, Netherlands Comprehensive Cancer Organisation, Eindhoven, the Netherlands; w Division of Psychosocial Research & Epidemiology, The Netherlands Cancer Institute, the Netherlands; x CoRPS - Center of Research on Psychological and Somatic Disorders, Department of Medical and Clinical Psychology, Tilburg University, Tilburg, the Netherlands

**Keywords:** Cancer survivorship, eHealth, Web-based intervention, Self-management, Health-related quality of life

## Abstract

**Introduction:**

The web-based self-management application Oncokompas was developed to support cancer survivors to monitor health-related quality of life and symptoms (Measure) and to provide tailored information (Learn) and supportive care options (Act). In a previously reported randomised controlled trial (RCT), 68% of 655 recruited survivors were eligible, and of those 45% participated in the RCT. Among participants of the RCT that were randomised to the intervention group, 52% used Oncokompas as intended. The aim of this study was to explore reasons for not participating in the RCT, and reasons for not using Oncokompas among non-users, and the use and evaluation of Oncokompas among users.

**Methods:**

Reasons for not participating were assessed with a study-specific questionnaire among 243 survivors who declined participation. Usage was investigated among 320 participants randomised to the intervention group of the RCT via system data and a study-specific questionnaire that was assessed during the 1 week follow-up (T1) assessment.

**Results:**

Main reasons for not participating were not interested in participation in scientific research (40%) and not interested in scientific research and Oncokompas (28%). Main reasons for not being interested in Oncokompas were wanting to leave the period of being ill behind (29%), no symptom burden (23%), or lacking internet skills (18%). Out of the 320 participants in the intervention group 167 (52%) used Oncokompas as intended. Among 72 non-users, main reasons for not using Oncokompas were no symptom burden (32%) or lack of time (26%). Among 248 survivors that activated their account, satisfaction and user-friendliness were rated with a 7 (scale 0–10). Within 3 (IQR 1–4) sessions, users selected 32 (IQR 6–37) topics. Main reasons for not using healthcare options in Act were that the information in Learn was already sufficient (44%) or no supportive care needs (32%).

**Discussion:**

Main reasons for not reaching or using Oncokompas were no symptom burden, no supportive care needs, or lack of time. Users selected many cancer-generic and tumour-specific topics to address, indicating added value of the wide range of available topics.

## Highlights

• Main reason for not participating in the RCT was no interest in scientific research.
• Main reason for no interest in Oncokompas was wanting to leave the period of being ill behind.
• Main reason for not using Oncokompas was no symptom burden.
• Many cancer-generic and tumour-specific symptoms were chosen in Oncokompas.

## 1 Introduction

Cancer survivors are nowadays expected to manage effects of cancer treatment, adopt a healthy lifestyle in order to reduce or prevent late effects, and cope with psychological consequences (Greene et al., 2015; Howell et al., 2020; Jansen et al., 2015). Self-management of these effects and navigating through available care options is not just for highly motivated cancer survivors, but is becoming necessary for all cancer survivors (Howell et al., 2020). Web-based self-management interventions can have positive effects on health-related quality of life (HRQOL) and symptom burden in cancer patients (Berry et al., 2014; Fridriksdottir et al., 2018; Howell et al., 2017; Skolarus et al., 2019; Warrington et al., 2019), and have the advantage that content can be tailored to the individual user, and are available at relatively low costs (Bashshur et al., 2013; Murray, 2012). However, knowledge is scarce on who is reached by such interventions (i.e. who is eligible for such interventions, and who participates in such interventions), and who uses those interventions as intended.

The web-based self-management application Oncokompas was developed to support cancer survivors in self-management, and contains three components: 1) measure: monitoring health-related quality of life and cancer-generic and tumour-specific symptoms by means of patient-reported outcome measures (PROMs), 2) learn: providing tailored information based on PROM scores, and 3) act: providing a personalized overview with recommended supportive care options (van der Hout et al., 2017). A randomised controlled trial (RCT) showed that Oncokompas is effective to reduce symptoms and improve HRQOL in cancer survivors (van der Hout et al., 2020b), and is not more expensive than usual cancer survivorship (van der Hout et al., 2020a). These are important conditions in order to implement Oncokompas in routine cancer survivorship care. However, to tailor implementation strategies it is also important to know which cancer survivors are reached and reasons why people are not reached, and to evaluate the actual usage of Oncokompas and reasons why cancer survivors are not using Oncokompas.

In our previous report, we investigated the reach by assessing the eligibility rate and participation rate, in the context of an RCT on the efficacy and cost-utility of Oncokompas (van der Hout et al., 2020b, van der Hout et al., 2020a, van der Hout et al., 2017). We found that 68% of the respondents were eligible to use Oncokompas (they had access to the internet and an e-mail address), of whom 45% agreed to participate in the RCT on Oncokompas. Factors associated with eligibility were male sex, younger age, higher health literacy, higher positive adjustment to cancer, no unmet needs regarding health system information and supportive care, and tumour type. Factors associated with participation were a medium and higher education level, unmet supportive care needs for sexual problems, and a higher belief of control of health by powerful others (van der Hout et al., 2020b). However, specific reasons why eligible people decided not to participate in this RCT are not known. As there is evidence on reasons why cancer survivors decline participation in clinical trials (Jenkins and Fallowfield, 2000; Mills et al., 2006; Naidoo et al., 2020; Viljoen et al., 2020), we focussed on reasons why eligible survivors were not interested in web-based self-management applications.

It is known that eHealth applications are not always used as intended (Kelders et al., 2012; Sieverink et al., 2017b). eHealth literacy and internet skills are important factors in using eHealth interventions as intended by the developers, and to profit from eHealth interventions (Halwas et al., 2017). Initial results on the usage of Oncokompas showed that 52% of the users, used Oncokompas as intended (van der Hout et al., 2020b). Usage as intended was defined as the minimal use that was expected to improve outcomes, and was defined as completing at least the components Measure and Learn for at least one topic. Factors that were found to be associated with usage as intended were a higher education level, having a partner, and not being employed (van der Hout et al., 2020b). Reasons for not using Oncokompas among those who did not use Oncokompas, as well as the evaluation of Oncokompas among users may provide more insight into how to improve usage of web-based self-management interventions.

The aim of this study was to investigate the usage of Oncokompas, by investigating reasons for not using Oncokompas, and to investigate system data and evaluate Oncokompas among users. The results are important in the continuous cycle of improvement and updating the content and design of web-based self-management interventions (Catwell and Sheikh, 2009; Granja et al., 2018).

## 2 Methods

### 2.1 Study design

The study was conducted in the context of an RCT on efficacy and cost-utility of Oncokompas. Details of the study procedures are described elsewhere (van der Hout et al., 2020b, van der Hout et al., 2017). To investigate the reach, a two-step inclusion method was used: a survey on supportive cancer care (part 1), and the actual RCT (part 2). Respondents of the survey were invited to participate in the RCT if they were eligible to use Oncokompas. They were eligible when they had internet access and an e-mail address (subsample 1). Via this two-step inclusion method associations of eligibility and participation could be investigated, because information on non-eligible survivors and non-participants was available from the survey. The study protocol was approved by the Medical Ethics Committee of the VU University Medical Center (2015.523), published previously (van der Hout et al., 2017), and registered in the Netherlands Trial Register (NTR5774). All participants provided (online) written informed consent.

Cancer survivors were invited via this two-step inclusion method from October 2016 until July 2017 by a letter from their (former) treating physician. From July 2017 to May 2018, 1462 cancer survivors were invited to participate directly. In total, 625 survivors participated in the RCT, of whom 320 were randomised into the intervention group, and had access to Oncokompas (subsample 2).

To investigate the usage of Oncokompas, system data was extracted from RCT participants randomised into the intervention group, who had access to Oncokompas (subsample 2). To evaluate the usage of Oncokompas, data from the first follow-up assessment in the RCT was used. The link to the follow-up assessment was sent by mail, 1 week after Oncokompas was used as intended. If Oncokompas was not used, the link was sent 2 weeks after randomisation.

### 2.2 In- and exclusion criteria

The inclusion criteria for the survey (part 1) were: being diagnosed with breast cancer, colorectal cancer, head and neck cancer or lymphoma; being ≥18 years, and having completed treatment with curative intent 3 months to 5 years ago (all treatment modalities). Cancer survivors who had not yet completed endocrine therapy or immunotherapy were included 3 months to 5 years after their previous treatment, and patients diagnosed with lymphoma who had a wait-and-see regimen, were included 3 months to 5 years after the date of diagnosis. The exclusion criteria for part 1 were: male cancer survivors diagnosed with breast cancer, severe cognitive impairment, insufficient mastery of the Dutch language, or physical inability to complete a questionnaire (e.g. blind, or paralyzed). Additional eligibility criteria for the RCT (part 2) were: having access to the internet and having an e-mail address.

### 2.3 Intervention

A detailed description of Oncokompas has been published previously (van der Hout et al., 2020b, van der Hout et al., 2017). In short, Oncokompas is an eHealth self-management application that supports cancer survivors to monitor their HRQOL and cancer-generic and tumour-specific symptoms. Oncokompas includes 32 topics in 5 cancer-generic domains: physical, psychological and social functioning, lifestyle, and existential issues. In addition, tumour-specific modules are available targeting head and neck cancer (6 topics), colorectal cancer (8 topics), breast cancer (9 topics) and (non-)Hodgkin lymphoma survivors (7 topics). Oncokompas consists of three components: ‘Measure’, ‘Learn’, and ‘Act’. In the ‘Measure’ component, cancer survivors complete PROMs on the topic(s) of choice. Data from the ‘Measure’ component are processed in real-time and linked to feedback in the ‘Learn’ component. In the ‘Learn’ component feedback is provided by means of a 3-colour system: green (no elevated well-being risks), orange (elevated well-being risks), and red (seriously elevated well-being risks). Cancer survivors receive personalized information on the outcomes (Learn, information), and comprehensive self-care advice (Learn, advice). In the ‘Act’ component, cancer survivors obtain a personalized overview with supportive care options. If a user has elevated well-being risks (orange score), the feedback includes suggestions for self-help interventions, and if a user has seriously elevated well-being risks, the feedback includes suggestions for medical specialists or their general practitioner (van der Hout et al., 2020b, van der Hout et al., 2017).

### 2.4 Outcome measures

#### 2.4.1 Reasons for not participating

Among eligible cancer survivors who were not willing to participate in the RCT (subsample 1), reasons for not participating were assessed in an online form with response options: ‘not interested in Oncokompas’, ‘not interested in scientific research’, ‘not interested in Oncokompas and scientific research’, and ‘other reasons’. Reasons for not being interested in Oncokompas were further explored with pre-set response options, and multiple reasons were allowed. In case the online form was not completed, but the reason for not participating was indicated by phone or e-mail, the reasons were categorized into one of the categories manually.

#### 2.4.2 Reasons for not using Oncokompas

Among all participants of the RCT, who were randomised to the intervention group (access to Oncokompas), but who did not use Oncokompas, reasons for not using Oncokompas were explored with pre-set response options in the first follow-up (T1) assessment of the RCT (1 week after usage of Oncokompas, with a maximum of 2 weeks after randomisation).

#### 2.4.3 Usage of Oncokompas

Usage was evaluated among those who were randomised into the intervention group in the RCT (subsample 2), and reported previously (van der Hout et al., 2020b). Usage was investigated via system data from Oncokompas and data from an evaluation questionnaire. System data was extracted separately for each component of Oncokompas (Measure, Learn and Act). For the component Measure: number of completed topics per user, and number of completions per topic; for the component Learn: number of green (no elevated well-being risk), orange (elevated well-being risk) and red scores (seriously elevated well-being risk) per user, and the number of green, orange, and red scores per topic, and for the component Act: number of clicks to supportive care options.

Usage as intended was defined as the completion of the components Measure and Learn for at least one topic, at least once during the 6-month follow-up period. Based on expert opinion of our team, with experience from clinical practice, together with directions from literature, it was expected that using at least these two components are needed to improve outcomes. Studies have shown that PROMs can be used for screening to identify symptoms, and can be used to track changes over time (Duman-Lubberding et al., 2017; Snyder et al., 2011; Velikova et al., 2008). Clinical experience suggest that completing PROMs is already beneficial for cancer survivors, but evidence is lacking (Coyne, 2013; Palmer et al., 2011), and therefore, the information in the component Learn is thought to be needed for an actual beneficial effect. The component Act is possibly not needed for each user, as the information and self-care advice can already be sufficient to improve the outcomes. The usage rate was calculated as the number of users who used Oncokompas as intended, divided by the total number of users.

In the T1 assessment, participants were invited to complete the study-specific evaluation questionnaire. Satisfaction and user-friendliness were evaluated via items on an 11-point rating scale (0−10). User experiences and satisfaction on several aspects of the components Measure, Learn and Act were evaluated via multiple-choice questions.

### 2.5 Statistical analyses

Descriptive statistics were generated to characterize the study sample (by means of frequency and percentage for categorical data and median and interquartile range (IQR) for continuous data), calculate the eligibility, participation and usage rates and describe the reasons for not participating and not using Oncokompas, and system data. Statistical analyses were performed using IBM SPSS Statistics version 26 (IBM Corp., Armonk, NY, USA).

## 3 Results

**Fig. 1:**
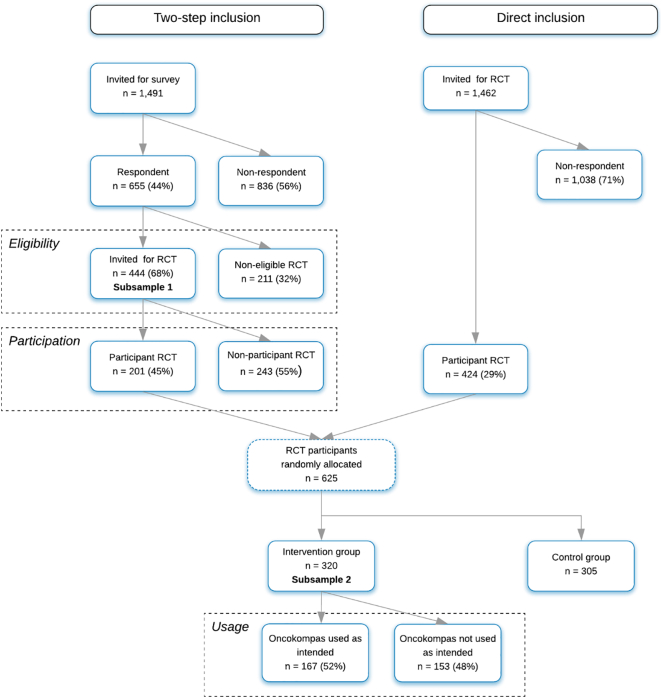
Flow chart of the study.

The flow chart of the study is shown in Fig. 1. Characteristics of the study population are shown in Table 1.

**Table 1:** Baseline characteristics of cancer survivors analysed for the reach and usage.

Characteristics^a^	Reach (subsample 1; *n* = 444)		Usage (subsample 2; *n* = 320)	
	Non-participants of RCT (*n* = 243)	Participants of RCT (*n* = 201)	Non-users (*n* = 153)	Users as intended (*n* = 167)
Age (years)	65 (57–71)	64 (56–70)	65 (55–72)	65 (57–70)
Sex (female)	148 (61%)	120 (60%)	73 (48%)	85 (51%)
Marital status (partner)	193 (79%)	174 (87%)	120 (78%)	144 (86%)
Education level				
Low	110 (46%)	67 (33%)	59 (39%)	52 (31%)
Medium	64 (26%)	71 (35%)	54 (35%)	51 (31%)
High	68 (28%)	63 (31%)	40 (26%)	63 (38%)
Employment status (employed)	75 (31%)	69 (34%)	66 (43%)	56 (34%)
Tumour type				
Breast cancer	88 (36%)	82 (41%)	35 (23%)	31 (19%)
Colorectal cancer	73 (30%)	61 (30%)	41 (27%)	39 (23%)
Head and neck cancer	45 (19%)	34 (17%)	40 (26%)	59 (35%)
Lymphoma	37 (15%)	24 (12%)	37 (24%)	38 (23%)
Tumour stage				
Stage I	73 (30%)	77 (38%)	52 (34%)	54 (32%)
Stage II	64 (26%)	44 (22%)	31 (20%)	42 (25%)
Stage III	53 (22%)	40 (20%)	28 (18%)	33 (20%)
Stage IV	28 (12%)	26 (13%)	32 (21%)	32 (20%)
Unknown	25 (10%)	14 (7%)	10 (7%)	6 (4%)
Time since diagnosis				
3-<12 months	29 (12%)	22 (11%)	23 (15%)	16 (10%)
12-<24 months	90 (37%)	66 (33%)	50 (33%)	54 (32%)
24-60 months	124 (51%)	113 (56%)	80 (52%)	97 (58%)
Treatment type (multimodal)	154 (64%)	140 (70%)	90 (60%)	93 (56%)
Comorbidities (multiple)	55 (23%)	48 (24%)	37 (24%)	34 (20%)

a Median (IQR), or n (%).

### 3.1 Reasons for not participating

Of the 444 eligible cancer survivors invited to participate in the RCT on Oncokompas (subsample 1), 201 agreed to participate (participation rate: 45%).

Of the 243 eligible survivors that declined participation, 152 (63%) responded and actively declined, and 91 (37%) did not respond. Among those who responded, reasons for not participating were they were not interested in participation in scientific research (*n* = 61, 40%), they were not interested in participation in scientific research and not interested in Oncokompas (*n* = 42, 28%), they were not interested in Oncokompas (*n* = 28, 18%), they did not want to participate because of personal reasons (*n* = 13, 9%), or they did not want to provide reasons (*n* = 8, 5%).

In total, 70 (29%) indicated that they were not interested in Oncokompas. Reasons for not being interested in Oncokompas (*n* = 70) was that someone wanted to leave the period of being ill behind (*n* = 20, 29%), did not experience any symptom burden (*n* = 18, 23%), thought they would lack sufficient internet skills (*n* = 14, 18%) no need for supportive care (*n* = 11, 14%), Oncokompas does not fit to their personal situation (*n* = 10, 13%), no time/motivation (*n* = 8, 11%), too confronting (n = 8, 11%), no need for information and advice (n = 7, 9%). No one indicated that the aim of Oncokompas was unclear (*n* = 0, 0%).

### 3.2 Reasons for not using Oncokompas

Among the 320 participants (subsample 2) who were randomised to the intervention group, 72 (23%) did not activate their account. Among them, 31 (43%) completed the first assessment of the RCT, two weeks after being provided access to Oncokompas. Among them, reasons for not using Oncokompas were no symptom burden (*n* = 10, 32%), lack of time (n = 8, 26%), not interested (*n* = 3, 10%), not fitting to personal problems (n = 3, 10%), personal reasons (n = 3, 10%), login details lost or not received (n = 3, 10%), forgotten to activate Oncokompas account (*n* = 2, 6%), aim of Oncokompas was not clear (n = 2, 6%), too difficult (n = 1, 3%), too comprehensive (n = 1, 3%), technical problems (n = 1, 3%). No one indicated that it was too confronting.

### 3.3 Usage of Oncokompas

**Fig. 2:**
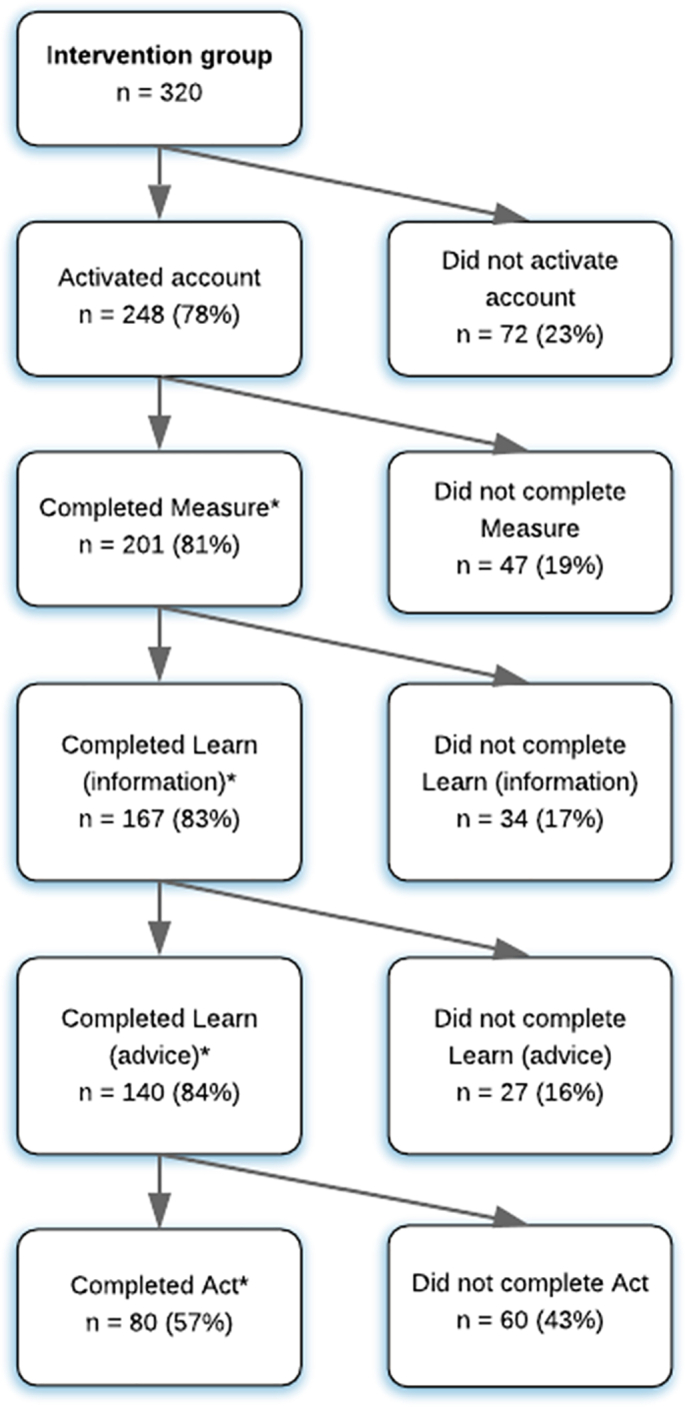
Flow chart of usage of Oncokompas. * for at least 1 topic.

Among the 320 participants (subsample 2) who were randomised to the intervention group, 248 (78%) activated their account, and 167 (52%) used Oncokompas as intended (usage rate: 52%) (van der Hout et al., 2020b). The flow chart of the usage, with the completion per subsequent component is shown in Fig. 2.

During the 6-months follow-up period of the RCT, the median number of logins in Oncokompas was 3 (IQR 1–4) among the 248 who activated their account, and 3 (IQR 2–5) among the 167 who used Oncokompas as intended.

Among the 248 participants who activated their Oncokompas account, 217 (88%) completed the first follow-up assessment (one week after using Oncokompas for the first time). The median score on satisfaction was 7 (IQR 6–8), and on user-friendliness 7 (IQR 5–8). The median score on the question ‘How likely is it that you will recommend Oncokompas to other cancer survivors’ was 6 (IQR 5–7). Self-reported time spent in Oncokompas was less than 30 min as reported by 28%, between 30 and 60 min by 48%, and more than 60 min by 23% of the users. The time it took to complete Oncokompas was evaluated as ‘way too long’ by 6%, ‘too long’ by 25%, ‘exactly right’ by 66%, and ‘too short’ by 3% of the users. A minority (15%) indicated that they had help of others (e.g. partner) using Oncokompas, and none of the participants reported that they e-mailed or called the helpdesk of Oncokompas. Most users (75%) intended to login to Oncokompas again and read the information and advice, and supportive care options once again. Most users (71%) indicated that they wanted to use Oncokompas again.

#### 3.3.1 Measure

**Fig. 3:**
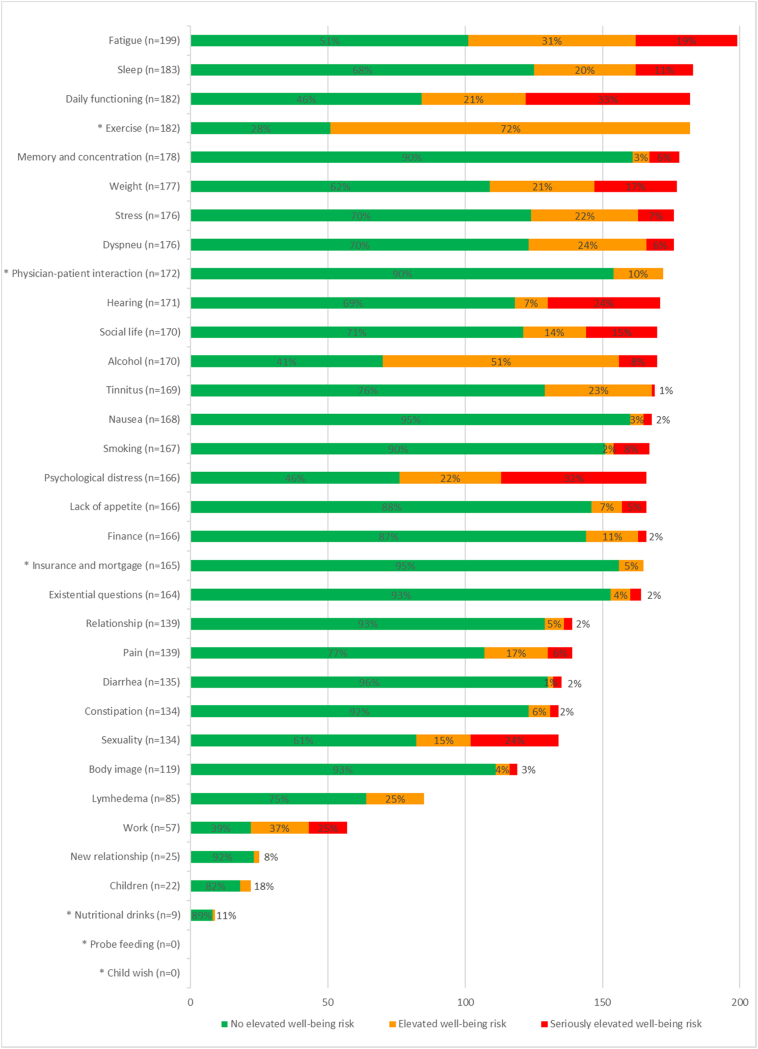
Number of completions per cancer-generic topic within Oncokompas, and corresponding scores, based on system data. * on these topics users can only score green or orange.

In total, 201 participants (81% of those who activated their account) completed the component Measure for at least 1 topic (Fig. 2), during the 6-months follow-up period. The median number of topics completed per person was 32 (IQR: 6–37). The cancer-generic topics that were chosen most often were: fatigue, sleep, and daily functioning, all from the physical quality of life domain (Fig. 3).

The number of questions in the Measure component was rated as ‘not feasible’ by 6%, ‘a little feasible’ by 41%, ‘feasible’ by 41%, and ‘very feasible’ by 9%. The overlap between questions in Measure was rated as ‘not’ by 16%, ‘a little’ by 57%, ‘much’ by 24%, and ‘very much’ by 3%.

#### 3.3.2 Learn

**Fig. 4:**
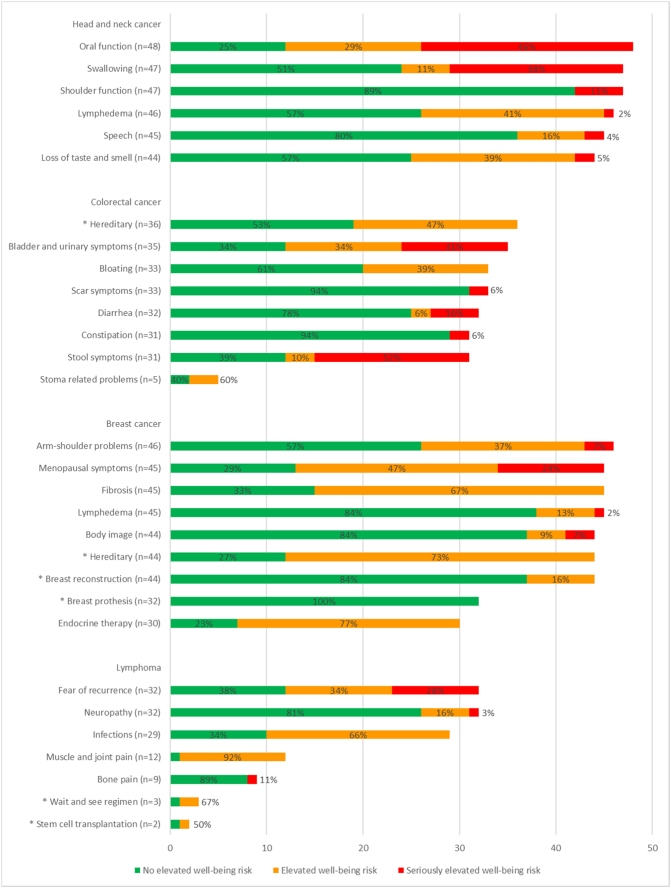
Number of completions per tumour-specific topic within Oncokompas, and corresponding scores, based on system data. * on these topics users can only score green or orange.

In total, 167 users (83% of those who completed the Measure component) read the page with information for at least 1 topic. Of them, 140 users (84% of those who read the information page) also read the page with advice and self-help tips for at least 1 topic. In total, 4497 topics were completed, on which 73% of the users had a green score, 18% had an orange score, and 9% had a red score. Per user, the median number of green scores was 21 (IQR 2–28), the median number of orange scores was 4 (IQR: 2–8), and the median number of red topics was 2 (IQR: 0–4). The scores on cancer-generic topics are shown in Fig. 3 and the scores on tumour-specific topics are shown in Fig. 4.

The question ‘Did the score correspond with your personal experience?’ was answered as ‘not corresponding’ by 7%, ‘little corresponding’ by 43%, ‘much corresponding’ by 45%, and ‘very much corresponding’ by 5% of the users. Most users (74%) rated the scores as clear and understandable. The information accompanying the scores was rated by most users as clear (72%), complete (63%), and useful (53%). Of the users, 61% indicated that the provided information and advice in the Learn component did not fit their personal situation, 57% that it not fit their health status, and 35% indicated that the information had added value for them. Slightly more than half (52%) of the users indicated that they received sufficient information to cope with their problem.

#### 3.3.3 Act

In total, 80 users (57% of those who completed the Learn component) completed the Act component for at least 1 topic (Fig. 2). The number of proposed supportive care options was rated as ‘too little’ by 13%, ‘exactly right’ by 66%, and ‘too much’ by 21% of the users.

Nineteen percent of the users indicated that they had already used supportive care options suggested by Oncokompas after 1-week follow-up, and 41% of the users indicated that they wanted to use the proposed supportive care options in the near future.

Seventy-two users indicated that they did not want to use the proposed supportive care options in the Act component. Reasons were that the information and advice provided in Oncokompas was already sufficient (*n* = 32, 44%), they were not interested or did not have a need for the supportive care option (*n* = 23, 32%), they were already using the supportive care option (*n* = 10, 14%), they used the supportive care option before (*n* = 9, 13%), they were limited in functioning, therefore using supportive care options was not possible (*n* = 6, 8%), a lack of time (*n* = 5, 7%), there was too little information on the supportive care option available (n = 3, 4%). No one indicated that the supportive care option was not available or had a wait-list, the supportive care option was too far away, or the supportive care option was too expensive and/or was not reimbursed.

## 4 Discussion

In this study, we investigated reasons for not reaching or not using the web-based self-management application Oncokompas among cancer survivors, and evaluated Oncokompas among users. Half of the eligible cancer survivors did not want to participate in the RCT investigating Oncokompas. The most often indicated reason for not participating in the RCT was that cancer survivors did not want to participate in scientific research (40%), followed by that they did not want to participate in scientific research and were not interested in Oncokompas (28%), and were not interested in Oncokompas (18%). Main reasons for not being interested in Oncokompas were that cancer survivors wanted to leave the period of being ill behind, did not experience symptom burden, thought they would lack sufficient internet skills, or they did not have a need for supportive care. The main reason for not using Oncokompas among RCT participants was that no symptom burden was experienced.

Among breast cancer survivors, the RCT participation rate was highest, but actual Oncokompas usage rate was lowest compared to the other tumour types. It might be that breast cancer survivors are willing to participate in scientific research, but that they are performing relatively well, and therefore did not use Oncokompas as often. In contrast, head and neck cancer survivors were participating less frequently in the RCT, but if they did, they used Oncokompas more often, possibly because they were suffering from symptoms more often. This corresponds with the percentage of seriously elevated well-being risks on tumour-specific symptoms, which was lowest among breast cancer survivors (5% of completed tumour-specific topics), and highest among head and neck cancer survivors (18% of completed tumour-specific topics).

While reasons such as no symptom burden or no supportive care needs are legitimate reasons for not participating or using Oncokompas, other reasons mentioned might be useful to improve (access to) web-based interventions. These include no time, not fitting to personal situation and that people think they lack skills to use such an intervention. These reasons emphasize the need for easy to use applications, with simple login procedures, which do not take much time to use. Tailoring evidence-based information to the individual cancer survivor is seen as an advantage of Oncokompas, as this makes the information applicable to the users' situation and needs, and can be directly applied (Murray, 2012). However, further tailoring might improve Oncokompas, as more than half of the users indicated that the information did not match with their personal situation and health status, and one third of the users indicated that it took too much time to complete Oncokompas.

The number of topics that users chose to address during 6-months follow-up was high (median of 32 topics, during a median of 3 sessions). This may explain why one third of the users rated the time it took to complete Oncokompas as too long. Encouraging users to address one or two topics at a time, that are the most important for them, and stimulate repeated use to cover multiple topics is recommended to improve usage as intended. Users were burdened by many different cancer generic, as well as tumour-specific symptoms, which is in line with studies on symptoms that cancer survivors can experience (Aaronson et al., 2014; Given and Given, 2013; Harrington et al., 2010; Rowland and Bellizzi, 2014). Furthermore, symptoms should be seen as a cluster of interrelated symptoms, and not seen as isolated symptoms (Aaronson et al., 2014). Therefore the range of topics within web-based self-management interventions should be wide, and not focussed on only one topic.

Multimedia tools such as podcasts, videos, infographics and gamifications elements may also increase usage and stimulate repeated use (Araújo-Soares et al., 2019; Kelders et al., 2013; Moore et al., 2019; Perski et al., 2017; Sardi et al., 2017; Sieverink et al., 2017b). Moreover, instructions for healthcare professionals on how to recommend Oncokompas to cancer survivors might increase the reach.

Users had no elevated well-being risks on 73% of the topics they completed and had (seriously) elevated well-being risks on 27% of the topics. The cancer-generic topics that were most often selected were fatigue, sleep, and daily functioning, all from the physical quality of life domain. Topics with the highest percentage of seriously elevated well-being risk (red score) were daily functioning, psychological problems, work, and sexuality, which are symptoms often observed among cancer survivors (Aaronson et al., 2014; Eeltink et al., 2021; Foster et al., 2009; Harden et al., 2015; Stein et al., 2008). Whereas some other web-based self-management interventions target a single topic, or a limited amount of topics (Foster et al., 2016; Kim and Park, 2015; Willems et al., 2015), we think that the variety of topics that are incorporated in Oncokompas is valuable in self-management of HRQOL and symptoms, because of the wide range of symptoms that cancer survivors can experience (Aaronson et al., 2014; Given and Given, 2013; Lagergren et al., 2019; Stein et al., 2008). A previous qualitative study among head and neck cancer patients showed that some participants had doubts about the added value of Oncokompas, but they mentioned it would be helpful when symptoms are present (Duman-Lubberding, 2018) When there is a wide range of available topics, there is more chance that it is applicable to the users situation.

It was found that after each step of Oncokompas (Measure, Learn, Act), some users were not going to the next step. After activation of the account 19% did not go to the Measure component, 17% of them did not go to the Learn component with information, 16% of them did not go the Learn component with advice, and 43% of them did not go to the Act component. About half of the users indicated that the information and advice provided in the component Learn was already sufficient to cope with their problem, and therefore, the component Act might not be necessary for all users. Oncokompas is a complex intervention, with multiple components, domains and topics, and every cancer survivor has other preferences and needs. Therefore, it is difficult to measure the dose-response relation of usage, and determine the accurate cut-off point when it is used as intended (Murray, 2012; Sieverink et al., 2017a). In this study, we used the definition of used as intended when the components Measure and Learn were completed for at least one topic. Only 52% of the participants met the defined criteria, which is similar to other studies, showing that about 50% of the participants fully adhere to web-based interventions (Kelders et al., 2012). Reasons for non-use were similar to those of a scoping review on non-using digital patient-reported outcome interventions, that reasons were related to skills, emotional distress and technical barriers (Nielsen et al., 2020).

Among users, Oncokompas was evaluated positively and most users indicated that they wanted to use Oncokompas again. In contrast, only 35% of the cancer survivors reported that the information had added value for them, and the question whether they would recommend Oncokompas to other cancer survivors was rated with a median of 6. This seems contradictory to the 71% of users that indicated that they want to use Oncokompas again. This might suggest that knowing that support is available when needed, or when symptoms are present is already sufficient. Further research into usage patterns is needed to gain insight into which specific components and topics contribute to the intervention effect of Oncokompas, and to be able to predict which user needs which components (Jonkman et al., 2016). Usage patterns would be helpful to gain insight into ways to improve usage (Kelders and Van Gemert-Pijnen, 2013).

A limitation of this study is that we used ‘willing to participate in an RCT on Oncokompas’ as a proxy for ‘being interested in Oncokompas’, while these measures might not correspond in practice. Most of the non-participants indicated they were not interested in participation in scientific research (40%), but there is also a considerable number of non-participants that indicated they were both not interested in scientific research and in Oncokompas (28%). Unfortunately, no additional (qualitative) data is available to know which was the main reason for declining participating in the RCT. Often mentioned reasons for declining participation in clinical cancer trials, e.g. in medication trials, are that cancer patients did not want to get randomised, did not want to get a placebo, or fear related to the clinical trial (Jenkins and Fallowfield, 2000; Mills et al., 2006; Naidoo et al., 2020; Viljoen et al., 2020). It could be questioned whether these reasons are similar for declining participation in RCTs on eHealth interventions, as these interventions are relatively low intensive. Reasons for declining participation in trials with those types of interventions might be that they lack skills, are unable to engage with those interventions or did not perceive a need for it (Foster et al., 2015). Another limitation is that the findings reported per tumour type are based on relatively small study samples. Further research on real-world data, and qualitative research is necessary to extend our knowledge on the usage of web-based self-management applications as Oncokompas. The scientific context in which Oncokompas was offered to cancer survivors, might have led to selection bias, and the results might have been different when offered in routine care. We found that the scientific context plays a role in the decision not to participate in web-based self-management interventions, as this was the main reason for not participating. When Oncokompas was offered directly to cancer survivors, the response rate was lower than the response rate of the survey of supportive care (step 1) (29% vs. 44%). Furthermore, respondents of the survey were older and had a shorter time since diagnosis than non-respondents of the survey, but there were no differences regarding sex, tumour type, or tumour stage (van der Hout et al., 2020b). However, due to ethical and practical reasons, we think this was the best method to investigate the reach of Oncokompas.

In conclusion, reasons for not reaching or using Oncokompas were no symptom burden, no need for supportive care, or lack of time. The main lessons learned are that eHealth applications aiming to support cancer survivors to improve their quality of life and reduce symptoms, should encompass a large variety of topics that users can choose from, and that survivors seem most burdened by tumour-specific symptoms and therefore these topics should definitely be included. In order to improve the reach and use of web-based self-management interventions, they should be easy to with simple login procedures, which do not take much time to use.

## Funding

This work was supported by the 10.13039/501100004622Dutch Cancer Society (KWF Kankerbestrijding) (grant number VU 2014-7202).

## Declaration of competing interest

IMV-dL has received grants from the Dutch Cancer Society (KWF Kankerbestrijding), Pink Ribbon, the Netherlands Organization for Health Research and Development (ZonMW), the SAG Foundation–Zilveren Kruis Health Care Assurance Company, Danone Ecofund–Nutricia, Red-kite (distributor of eHealth tools), and Bristol-Myers Squibb, during the conduct of this study. CRL has received personal fees for global advisory board participation from MSD, during the conduct of this study. All other authors have no conflicts of interest.
